# Oxytocin, prostaglandin F2_α_, and scopolamine for uterine involution of dairy cows

**DOI:** 10.3389/fvets.2024.1405746

**Published:** 2024-05-02

**Authors:** Alice Carbonari, Matteo Burgio, Lorenza Frattina, Edmondo Ceci, Maurizio Sciannamblo, Pasquale Ricci, Vincenzo Cicirelli, Annalisa Rizzo

**Affiliations:** Department of Veterinary Medicine, University of Bari Aldo Moro, Valenzano, Italy

**Keywords:** bovine, oxytocin, prostaglandin F2_α_, scopolamine, uterine involution

## Abstract

The aim of the study was to compare the effect of three substances with ecbolic activity, Oxytocin, Prostaglandin F2α (PGF2α) and Scopolamine, on the uterine involution process in dairy cows and on the resumption of ovarian activity. Eighty bovine were randomly divided in four groups: GROUP C: 20 cows treated, within 24 h of calving, with 5 mL/head of saline solution; GROUP PG: 20 cows treated, within 24 h of calving, with 150 μg/head of d-cloprostenol; GROUP OX: 20 cows treated, within 24 h of calving, with 50 IU/head of oxytocin acetate; GROUP S: 20 cows treated, within 24 h of calving, with 40 mg/q Scopolamine Butylbromide. Each cow was subjected to blood samples to evaluate the Hydroxyproline (HYP) levels, at T0, within 24 h after calving, and T7, T14, T28, 7, 14, and 28 days after calving, respectively. At T14 and T28, an ultrasound examination was performed to measure the diameter of ex-pregnant horn. In all cows, the reproductive indices (days to first service and number of artificial insemination for conception) were evaluated. In all groups, the HYP concentrations have been rising from T0 to T28, with the maximum levels obtained at T28 in the groups PG and S. As regard the diameter of uterine horn, the comparison among the groups showed significant differences only at T28, with lower values in the group PG and S. In group S and PG, the days to first service were less than other groups. Treatment with Scopolamine and PGF2α resulted in better outcomes, evidenced clinically by more efficient uterine involution and faster ovarian recovery.

## Introduction

Post-partum (PP) in dairy cows is the period that most influences the productive and reproductive efficiency of the animal (1). To allow a new pregnancy occurs the female genital apparatus undergoes physiological changes, such as uterine involution, epithelial regeneration, elimination of bacteria and resumption of ovarian cyclicity (2). Uterine involution is characterized by a series of macroscopic, microscopic and molecular events. The macroscopic ones affect the volume and weight of the uterus, while the microscopic ones concern regeneration. These changes are characterized by the phenomena of necrosis and desquamation of the caruncles and regeneration of the endometrium, which lead to a decrease in the weight of the organ, from an average weight of 13 kg before delivery to 1 kg 30 days later (3). These events are intended to ensure that the uterus regains suitable conditions for a new gestation (2). Furthermore, the molecular changes characterized by the reconstitution of Ca^2+^ and glucose reserves are crucial for the proper resumption of uterine contractility. The latter, after an initial increase, undergoes a reduction in the frequency and number of contractions to about 14 per hour in the next few hours after delivery; in the next 42 h, contractile events must further reduce to 1 per hour to ensure that placental retention does not occur (4). Complete uterine involution is reached around 40–50 days PP (2). The number of days required for proper involution increases in cows that have experienced complications during parturition (e.g., dystocias) or retention of fetal membranes; in these cows the risk of developing a uterine infection increases, as the possibility of bacterial colonization is greater (5). Failure of the uterus and cervix to return to normal size, in particular a cervical diameter of 7.5 cm, between 20 and 33 days PP, is associated with a reduced rate of conception as reported by LeBlanc et al. (6). Several pharmacological approaches are used to condition PP, all of which focus on modulating the contractile activity of the uterus. Currently, ecbolic drugs such as oxytocin (OX) and natural or synthetic Prostaglandin F2α (PGF2α) analogs are widely used in cattle breeding (7–9). There are conflicting opinions on the use of such ecbolics. Treatment with OX has a positive effect on uterine contractility up to day 2 PP, given its low half-life (7). The administration of PGF2α has a positive effect on uterine involution (8, 9), promoting a rapid completion of the process and a rapid recovery of ovarian activity (10). Stephen et al. (11), on the other hand, compared the two ecbolics administered for 1 week postpartum and found no positive effects on uterine involution, incidence of endometritis and reproductive performance. The parasympathetic nervous system also regulates the contractile activity of the uterus, promotes its vascularization and stimulates secretion from the cervical glands (12, 13). These activities are mediated by acetylcholine, which binds to muscarinic receptors, M2 and M3. Binding to the M2 receptor prevents relaxation of the uterus, while binding to the M3 receptor promotes its contraction (14). The distribution of the receptors is regulated by the hormone most present: estrogens stimulate the synthesis of the M2 receptor, while reducing that of the M3 receptor. In the 24 h after delivery, estrogens are still present in high concentrations and, therefore, M2 receptors are the most prevalent muscarinic receptor subtypes (15). Rizzo et al. (16) evaluated the effect of an antimuscarinic (parasympatholytic) drug, scopolamine, administered within 24 h after calving, to regularize uterine contractions and, thus, improve uterine involution in dairy cows. Scopolamine has proved to be a valid alternative to traditional ecbolic substances in the management of PP in dairy cows, regularizing uterine contractility by blocking, for the duration of its half-life (2–3 h), uterine contractions in PP, which are then more efficient and regular when they resume (16). The aim of the present study was to compare the effect of three substances with ecbolic activity, Oxytocin, PGF2α and Scopolamine, administered within 24 h of calving, on the uterine involution process in dairy cows and on the resumption of ovarian activity. To compare the efficacy of these drugs, hydroxyproline (HYP) levels, an important marker of uterine involution (16), ultrasound examination, for the measurement of the diameter of the uterine horns and reproductive indices (days to first service and number of artificial insemination for conception) were evaluated.

## Materials and methods

All procedures were conducted in accordance with animal welfare and use guidelines, with the informed consent of the owner and approval of the ethics committee (protocol no. 10/2023).

### Animals

The study involved 80 Friesan dairy cows located on a farm in the province of Benevento, from 4 to 6 year old, with 550 kg mean weight (range: 520–600 kg), free from non-infectious and infectious diseases. The free-housed animals were fed by unifeed, composed of corn silage, oat hay, medical hay, corn flour, soybean meal, cotton, crushed barley, beet pulp, and vitamin and oligomineral supplements. All cows, prior to any experimental procedure, underwent a general and particular objective examination of the reproductive apparatus, by means of rectal exploration, to diagnose any pathologies. All the subjects examined had eutocic calving and had no retention of fetal membranes.

The cows were randomly divided into four groups:

- GROUP C: 20 cows treated, within 24 h of calving, with 5 mL/head of saline solution (NaCl 0.9%), in one I.M. administration;
- GROUP PG: 20 cows treated, within 24 h of calving, with 150 μg/head of d-cloprostenol (Dalmazin^®^- Fatro-Italy), equivalent to 2 mL/head, in one I.M. administration;
- GROUP OX: 20 cows treated, within 24 h of calving, with 50 IU/head of oxytocin acetate (Neurofisin^®^- Fatro-Italy), equivalent to 5 mL/head, in one I.M. administration;
- GROUP S: 20 cows treated, within 24 h of calving, with 40 mg/q Scopolamine Butylbromide (Spasmolax^®^- Fatro-Italy), equivalent to 2 mL/q, in one I.M. administration.

### Blood withdrawals

Each cow was subjected to blood samples to evaluate the HYP levels, at the following time points, in according to Rizzo et al. (16):

- T0: within 24 h after calving.
- T7: 7 days after calving.
- T14: 14 days after calving.
- T28: 28 days after calving.

Blood samples were taken from the coccygeal vein in serum vacutainer tubes and transferred to the laboratory (20 ± 10 min). The samples were centrifuged at 1,620 × g for 10 min at +4°C. The serum was stored in 1.5-mL Eppendorf tubes at −20°C until further analyses. ELISA kit (Bovine Hydroxyproline ELISA Kit MyBio Source Inc., California) was used to measure the serum HYP levels, by using the following manufacter’s instructions. The kit has a detection range of 2,000 to 31.2 ng/mL; a sensitivity such that the minimum detectable is greater than 12 ng/mL; a specificity such that no cross-reaction with other substances occurred; an intra-assay accuracy of ≤8%; and an inter-assay precision of ≤12%.

### Ultrasound examination

At each check-up, all cows underwent a particular objective examination of the reproductive apparatus, by means of rectal exploration, to assess the possible occurrence of metritis and ovarian function; during the checks carried out at T14 and T28, an ultrasound examination was also carried out to measure the diameter of the uterine horn that had received the previous pregnancy, to monitor the degree of uterine involution. Ultrasound examination was performed using a multifrequency linear probe (5–10 MHz, set at 7.5 MHz) (SonoSite MicroMaxx Bothell, WA, United States) and filter set to 100 Hz. Ultrasound was always carried out by the same expert technician. To reduce the interposition of air, cause of artifacts, the probe was placed in the finger of an examination glove with ultrasound gel, before the examination. Feces were removed from the rectum and the diameter of the ex-pregnant uterine horn was measured in the B-mode. At least three images of uterine horn were stored and on this each one a cross-sectional diameter (from serosa to serosa) was detected. The mean of three transverse diameters was calculated, for each bovine.

### Reproductive indices

In all the enrolled cows, the reproductive indices in the postpartum were evaluated: days to first service and number of artificial insemination (AI) for conception. Around the 40th day of the postpartum, a clinical visit, to evaluate the condition of the genital system, was performed. Estrus detection, oedema of the vulva, clear mucosal vaginal discharge, standing to be mounted were observed. Estrus was confirmed by the detection of a preovulatory follicle on transrectal palpation. At healthy heat, all the cows were inseminated with frozen semen of proven bulls, obtained from specialized centers and referenced for preparation. Artificial insemination was always carried out by the same operator. The cows that did not return to heat, 40 days after the AI, underwent a clinical examination, during which the diagnosis of pregnancy was confirmed by means of a trans-rectal ultrasound with a multifrequency linear probe (5–10 MHz, set at 7.5 MHz) (SonoSite, MicroMaxx Bothell, WA, United States). The number of AI for conception was determined based on the number of AIs to obtain a pregnancy, so at each return to heat the cows were reinseminated until pregnancy was diagnosed.

### Statistical analysis

The results were analyzed using the statistical program SPSS 19 (IBM, NY). The ANOVA test was used for comparison between groups, while the GLM test for repeated measures with LSD post-hoc test was used for comparison within groups. For all tests, statistically significant differences were considered for *p* < 0.05.

## Results

The administration of all drugs did not produce any side effects. In the group C, two cows reported an acute metritis; in the group PG one cow showed subacute metritis; in the group OX one cow reported the left abomasal displacement and two cows follicular cyst; in the group S, one cow showed follicular cysts. These cows were not included in the experimental study.

### Hydroxyproline level

**Figure 1:**
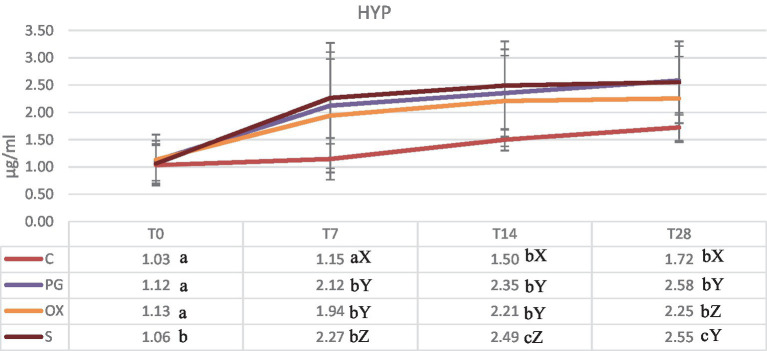
Concentrations (mean ± SD) (μg/mL) of serum hydroxyproline in groups C (control), PG (treated with d-cloprostenol), OX (treated with oxytocin acetate), S (treated with Scopolamine Butylbromide), at time T0 (within 24 h of delivery), T7, T14, and T28, at 7, 14, and 28 from delivery, respectively. Different letters in the same row show significant differences between means: ^a,b^*p* < 0.01. Different letters in the same column show significant differences between means ^X,Y,Z^*p* < 0.05.

The levels of the HYP in four groups are shown in Figure 1. The serum concentration are in agreement with reported in literature (16–18). The comparison among the four groups highlighted significant differences at T7, T14 and T28 among group C and the other experimental groups. In all groups, the HYP concentrations have been rising from T0 to T28, with the maximum levels obtained at T28 in the groups PG and S.

### Ultrasound examination

**Figure 2:**
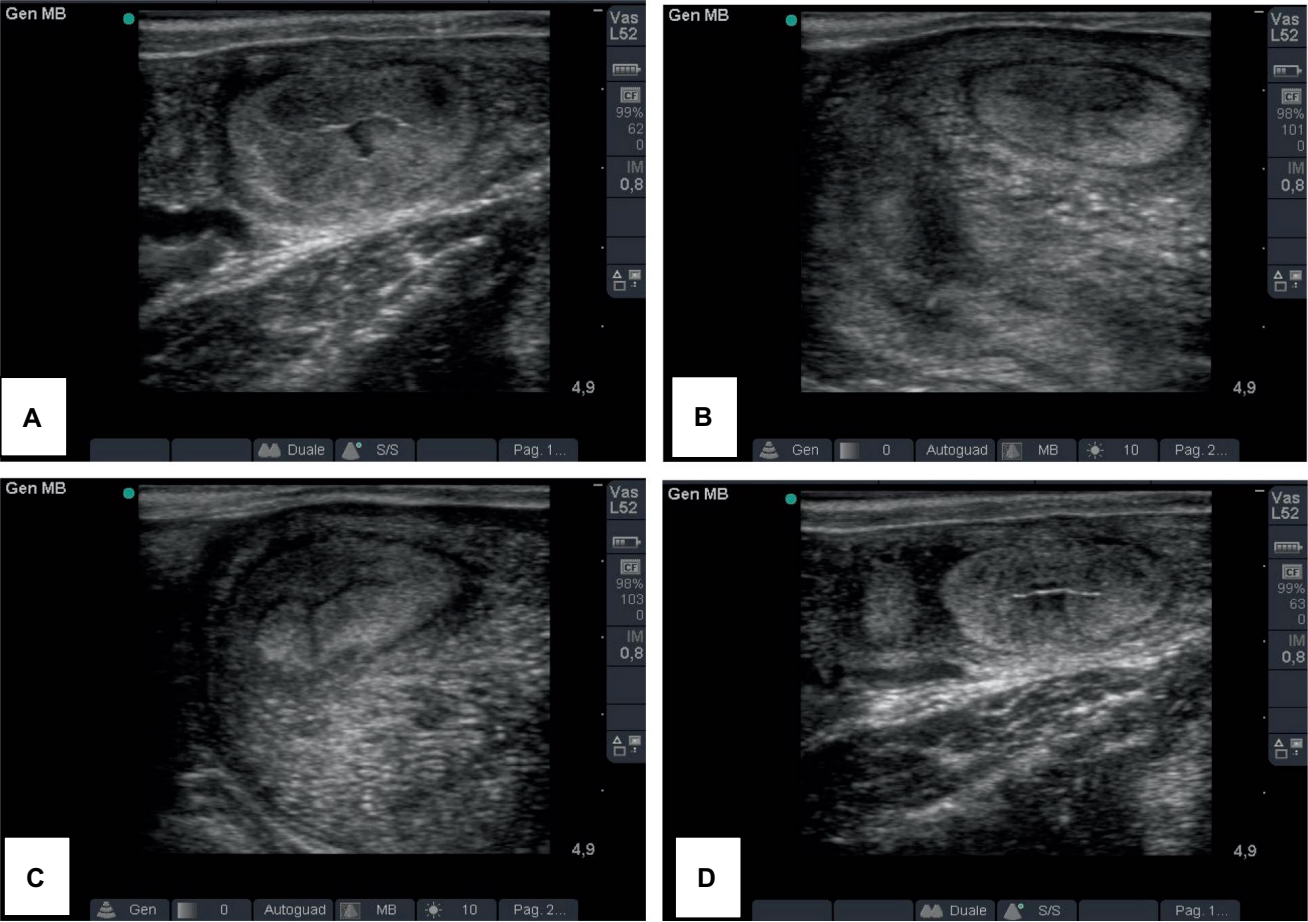
Representative ultrasound images of the ex-pregnant uterine horn of a cow of the S group **(A,B)** and a cow of the C group **(C,D)**, at T14 and T28, 14 and 28 days after delivery, respectively.

In the B-mode mode, the diameter of the ex-pregnant horn in the four groups are shown in Table 1. Representative ultrasound images of a cow of the S group, at T14 and T28 is showed in Figure 2. The mean diameter of the uterine horn decreased from T14 to T28, in all groups, in a statistically significant way. The comparison among the groups showed significant differences only at T28, with lower values in the group PG and S.

**Table 1:** Diameter (mean ± SD) (mm) of the ex-pregnant uterine horn in groups C (control), PG (treated with d-cloprostenol), OX (treated with oxytocin acetate), S (treated with Scopolamine Butylbromide), at time T14 and T28, 14 and 28 from delivery, respectively.

Groups	T14	T28
C	48.81 ± 5.41^a^	35.95 ± 4.20^Xb^
PG	44.78 ± 7.04^a^	28.36 ± 6.33^Yb^
OX	47.86 ± 9.14^a^	33.45 ± 5.96^Zb^
S	46.86 ± 2.24^a^	28.97 ± 2.86^Yb^

Different letters in the same row show significant differences between means: ^a,b^*p* < 0.001. Different letters in the same column show significant differences between means ^X,Y,Z^*p* < 0.05.

### Reproductive indices

The days to first service and number of artificial insemination are reported in Table 2.

**Table 2:** Reproductive indices (days to first service and number of artificial insemination -AI) in groups C (control), PG (treated with d-cloprostenol), OX (treated with oxytocin acetate) and S (treated with Scopolamine Butylbromide).

Groups	Days to first service	N° AI
C	49.9 ± 4.63^X^	3.2 ± 0.92^X^
PG	48.3 ± 3.95^X^	1.7 ± 0.67^Y^
OX	53.1 ± 4.75^Y^	2.7 ± 0.82^Z^
S	45.4 ± 2.95^Z^	1.8 ± 0.79^Y^

Different letters in the same column show significant differences between means ^X,Y,Z^*P* < 0.05.

## Discussion

The aim of the present study was to compare the effect of three substances with ecbolic activity, Oxytocin, PGF2α and Scopolamine, administered within 24 h of calving, on the uterine involution process in dairy cows and on the resumption of ovarian activity. The first two are conventional ecbolic drugs, commonly used, while scopolamine is an parasympatholytic drug that it was demonstrated to have ecbolic activity, regularizing uterine contractility (16). In present study, the efficacy of these drugs has been demonstrated by assaying HYP concentration, marker of uterine involution (16, 19). It increased from T0 to T28 in all groups, with concentrations lower in control group than the other groups. This confirm that the treatments with PGF2α, oxytocin and scopolamine, administrated within 24 h after calving, induced an improvement of uterine involution. They may have acted through their tonic effect on uterus, increasing the myometrial contractions and, consequently, improving the reduction of the organ and the clearance of intrauterine fluid (20, 21). HYP, in fact, is produced following to the uterine collagen fiber degradation, during the involution process (19). Among experimental groups, the higher HYP concentrations, at T28, are shown in groups PG and S. These results are also confirmed by those obtained with ultrasonography: in fact, the diameters of ex-pregnant uterine horn were reduced from T14 to T28, with values lower in groups PG and S than C and OX groups. It was hypothesizable that uterine involution was improved with PGF2α administration, as prostaglandins act not only on contractile activity but also on activation of the immune response and triggering of phagocytosis (9, 17). Moreover, prostaglandins act on contraction of cervical smooth muscle, promoting remodeling of the structure (22). Then, these activities can explain the higher HYP levels showed in group PG. In the S group, also, higher HYP concentrations were obtained. It is hypothesizable that, in this group, rebound effect was exploited, with positive influences on the involution of the organ, in agreement with Rizzo et al. (16). In other word, scopolamine temporarily has blocked the uterus contractions, for a period of time corresponding to its half-life (2–3 h). Following the disappearance of its pharmacological effect, the uterus has resumed contracting more effectively and increased glandular secretions, useful for self-cleansing activity. Moreover, it was demonstrated that the activation of muscarinic receptors M3 in muscle cells stimulates glucose uptake, essential element for the contractility (23). The activity on uterine involution by scopolamine could thus be attributed to both a regularization of contractility and an increase in contractile force due to increased glucose up-take. The efficacy of scopolamine on uterine involution was then demonstrated by both the high HYP levels and the reduction of the uterine horn diameters. In OX-treated subjects, on the other hand, there is an increase in the frequency of uterine contractions that promote the reduction of uterine volume, but immune stimulation is lacking. This would explain the lower HYP concentrations recorded in the OX group cows compared with the groups. Oxytocin, therefore, acting only on uterine contractility, was not as effective, in reducing diameters, as the other two drugs tested. In literature, Oxytocin and prostaglandin were used to influence the evolution of postpartum. However, there are conflicting opinions. Stephen et al. (11) compared the effect of oxytocin and dinoprost, administered for the first 7 days postpartum, on uterine involution, postpartum endometritis, and reproductive performance. The Authors concluded that these ecbolic drugs, as used in this study, were not recommended for use in clinical practice to improve involution or reproductive tract health in normal cows (11). On the other hand, Abdel-Khalek et al. (21) demonstrated that cows, treated with oxytocin or PGF2α within 6–12 h postpartum, showed similar results in terms of diameters of uterine horns (gravid and non-gravid), cervical and vaginal length than that occurred in the control cows. Moreover, the authors concluded that PGF2α and oxytocin treatment showed similar beneficial effects on uterine involution, meanwhile prostaglandin treatment obtained the best results on conception rate and number of services per conception within 90 days-post-partum (21).

As reproductive indices in present study, group S achieved the best results, in terms of days to first service that were less than other groups. It is known that the ovarian recovery is closely correlated with good uterine involution. Therefore, in this group, the improvement of the reproductive efficacy may be due to the scopolamine activity that regularize the uterine contractility but it also acts on vascularization (13) and on cervical glandular secretion (12). These activities are important for improving uterine clearance and, thus, allowing earlier resumption of reproductive function.

## Conclusion

The results of this study support the use of ecbolics in the immediate postpartum period to accelerate uterine involution and improve the reproductive efficiency of the dairy cow. Treatment with Scopolamine and PGF2α resulted in better outcomes, evidenced clinically by more efficient uterine involution and faster ovarian recovery.

## Data availability statement

The raw data supporting the conclusions of this article will be made available by the authors, without undue reservation.

## Ethics statement

All procedures were conducted in accordance with animal welfare and use guidelines, with the informed consent of the owner and approval of the ethics committee (protocol no. 10/2023). The studies were conducted in accordance with the local legislation and institutional requirements. Written informed consent was obtained from the owners for the participation of their animals in this study.

## Author contributions

AC: Conceptualization, Investigation, Methodology, Writing – original draft. MB: Data curation, Methodology, Writing – original draft. LF: Investigation, Methodology, Writing – original draft. EC: Investigation, Methodology, Writing – original draft. MS: Data curation, Writing – original draft. PR: Investigation, Writing – original draft. VC: Data curation, Methodology, Writing – original draft. AR: Conceptualization, Data curation, Methodology, Project administration, Supervision, Writing – original draft.
